# Influence of Caudovirales Phages on Humoral Immunity in Mice

**DOI:** 10.3390/v13071241

**Published:** 2021-06-26

**Authors:** Anton Chechushkov, Yuliya Kozlova, Ivan Baykov, Vera Morozova, Bogdana Kravchuk, Tatyana Ushakova, Alevtina Bardasheva, Ekaterina Zelentsova, Lina Al Allaf, Artem Tikunov, Valentin Vlassov, Nina Tikunova

**Affiliations:** 1 Laboratory of Molecular Microbiology, Institute of Chemical Biology and Fundamental Medicine Siberian Branch of Russian Academy of Sciences, 630090 Novosibirsk, Russia; achechushkov@gmail.com (A.C.); ulona@ngs.ru (Y.K.); ivan_baykov@mail.ru (I.B.); morozova@niboch.nsc.ru (V.M.); semali328@gamil.com (B.K.); ushakova@niboch.nsc.ru (T.U.); herba12@mail.ru (A.B.); allaflina@gmail.com (L.A.A.); arttik@ngs.ru (A.T.); vvlassov@ngs.ru (V.V.); 2 International Tomography Center Siberian Branch of Russian Academy of Sciences, 630090 Novosibirsk, Russia; zelentsova@tomo.nsc.ru; 3 Department of Natural Sciences, Novosibirsk State University, 630090 Novosibirsk, Russia

**Keywords:** bacteriophage, immunogenicity, antibodies, IgM, IgG, neutralization, humoral immunity, phage therapy, future medicine

## Abstract

Bacteriophages are promising antibacterial agents. Although they have been recognized as bacterial viruses and are considered to be non-interacting with eukaryotic cells, there is growing evidence that phages may have a significant impact on the immune system via interactions with macrophages, neutrophils, and T-cell polarization. In this study, the influence of phages of podovirus, siphovirus, and myovirus morphotypes on humoral immunity of CD-1 mice was investigated. In addition, tissue distribution of the phages was tested in these mice. No common patterns were found either in the distribution of phages in mice or in changes in the levels of cytokines in the sera of mice once injected with phages. Importantly, pre-existing IgM-class antibodies directed against capsid proteins of phages with myovirus and siphovirus morphotypes were identified in mice before immunization. After triple immunization of CD1-mice with phages without any adjuvant, levels of anti-phage serum polyclonal IgG antibodies increased. Immunogenic phage proteins recognized by IgM and/or IgG antibodies were identified using Western blot analysis and mass spectrometry. In addition, mice serum collected after immunization demonstrated neutralizing properties, leading to a substantial decrease in infectivity of investigated phages with myovirus and siphovirus morphotypes. Moreover, serum samples collected before administration of these phages exhibited some ability to reduce the phage infectivity. Furthermore, Proteus phage PM16 with podovirus morphotype did not elicit IgM or IgG antibodies in immunized mice, and no neutralizing activities against PM16 were revealed in mouse serum samples before and after immunization.

## 1. Introduction

In recent years, there has been an increase in the prevalence of infectious diseases caused by drug-resistant bacteria, and a more rapid increase is anticipated by the experts [1]. Subsequently, there is growing interest in the discovery of new therapies, particularly, bacteriophage-based antibacterial therapy. Important features of bacteriophages are their inability to infect eukaryotic cells and, unlike antibiotics, their lack of potential toxicity. In addition, bacteriophages are characterized by high specificity to the bacterial host, hence they are unlikely to influence normal intestinal microbiota [2]. Due to these factors, bacteriophages can be considered to be promising therapeutic agents with the potential to foster personalized treatment of severe antibiotic-resistant bacterial diseases [3].

Despite the apparent simplicity of bacteriophages, a large number of uncertainties remain regarding their interaction with the macro-organism. Administered orally, bacteriophages can easily penetrate intestinal epithelia, whereas when administered via the blood they can be distributed throughout tissues [4]. Bacteriophages can both stimulate and inhibit neutrophil activation [5], facilitate phagocytosis and adaptive immune responses [6,7,8], and possess prominent immunogenicity. Factors determining these interactions are unclear; however, phage-specific antibodies are of significant concern because they can influence phage infectivity by neutralization and Fc-dependent and complement-dependent phagocytosis [9,10]. Although anti-phage antibodies have been widely shown, their influence on therapy is unforeseen, demonstrating both worsening [11,12] and neutral [13] effects.

Recent data indicate bacteriophages can be captured and endocytosed by innate immune cells, such as neutrophils, macrophages, and dendritic cells [14,15]. Depending on specific molecular patterns, recognition of viral particles may be accompanied by cytokine production, increased cell-effector functions, activation of inflammatory cascade, and stimulation of T- and B-cell-mediated immune response. Some bacteriophages, such as PhiX174, induce strong T-cell-dependent immune response [5,6], and others, such as some *Escherichia coli* phages, mediate only moderate stimulation [16]. Generally, bacteriophages are now recognized to have immune-stimulating properties [17]; however, the exact qualities that mediate these effects are unexplained, because phages are typically not covered with glycoproteins and their nucleic acids are not exposed to the cytoplasmic milieu of eukaryotic cells [18]. 

The aim of this study is to investigate the influence of Caudovirales phages of different morphotypes on humoral immunity in vivo and their tissue distribution in mice.

## 2. Materials and Methods

### 2.1. Bacteriophages

In this study, *Proteus mirabilis* bacteriophages PM16 and PM135 (podovirus and siphovirus morphotypes, respectively), *Klebsiella* spp. bacteriophage KP179, and *Pseudomonas aeruginosa* bacteriophage PA136 (both from the *Myoviridae* family) were used (Table 1). Bacteriophages PM16, PM135, and KP179 were previously isolated from clinical samples and characterized at the Institute of Chemical Biology and Fundamental Medicine Siberian Branch of the Russian Academy of Science (ICBFM SB RAS), Novosibirsk, Russia [18,19]. Notably, KP179 was initially isolated against *Raoutella* *ornithinolytica* CEMTC 2544 and showed high lytic activity, not only against *R. ornithinolytica*, but also against *Klebsiella pneumoniae* and *Klebsiella oxytoca*. Bacteriophage PA136 was provided by Professor K. Miroshnikov (Institute of Bioorganic Chemistry RAS, Moscow, Russia).

The complete genomes of phages PM16, PM135, KP179, and PA136 have been sequenced, annotated [18,19], and deposited in the GenBank database with the accession numbers KM819694, MG030347, MH729874, and NC_041904.1, respectively. PM16, PM135, KP179, and PA136 phages are lytic, and genes encoding toxins or associated with lysogeny have not been found in their genomes.

### 2.2. Bacteriophage Propagation and Purification

Bacteriophages PM16 and PM135 were propagated using host strain *P. mirabilis* CEMTC 73; phages KP179 and PA136 were propagated using *K. oxytoca* CEMTC 2927 and *P. aeruginosa* CEMTC 1804, respectively. Bacterial cultures were obtained from the Collection of Extremophilic Microorganisms and Typical Cultures (CEMTC) of ICBFM SB RAS. A laboratory strain of *Escherichia coli* TG1 was used as a host for the propagation of phage M13K07 (New England BioLabs, Ipswich, MA, USA).

The exponentially growing bacterial culture (OD600 = 0.6) in a volume of 500 mL was infected with a suspension of the corresponding phage at a multiplicity of infection (MOI) of 0.1. The infected bacterial culture was incubated at 37 °C with shaking for 2–4 h until bacterial lysis began. *E. coli* TG1 was infected with phage M13K07 and grown overnight before phage harvest. Phage particles were precipitated from bacterial lysates using polyethylene glycol (PEG) and the phage precipitate was dissolved in phosphate buffer saline (PBS), with pH 8.0. The resulting phage suspension was purified from PEG and bacterial debris using a standard technique based on ultracentrifugation in a gradient of cesium chloride [20]. Dialysis against PBS, pH 8.0, was carried out and titers of viable bacteriophages were determined using a fresh layer of an appropriate bacterial host strain in the top agar. The obtained titers were 10^11^–10^12^ plaque-forming units/mL (PFU/mL). These purified phage preparations were further used for mass spectrometry. For other experiments, phage preparations were diluted in 0.9% NaCl (administration to mice) or PBS (ELISA) to a concentration of 10^9^ PFU/mL and 100 μL of phage suspension containing 10^8^ PFU of a distinct phage was used for administration to each mouse.

### 2.3. Bacteriophage Structural Proteins Analysis by SDS-PAGE and MALDI-TOF Mass Spectrometry

Proteins from purified preparations of phages PM135, KP179, and PA136 were separated using tris-glycine SDS 10% or 15% (*w*/*v*) polyacrylamide gel electrophoresis (SDS-PAGE) and visualized by Coomassie R250 staining. Gel fragments containing individual protein bands were cut from the appropriate gels and trypsin digestion was carried out, as described previously [21]. The obtained peptides were desalted using C18 ZipTips (Millipore, Burlington, MA, USA), mixed (in a ratio of 0.5 μL of the sample to 0.5 μL of the matrix) with 2.5-dihydroxybenzoic acid dissolved in 70% acetonitrile and 0.1% trifluoroacetic acid, and spotted on a standard MTP ground steel plate (Bruker Daltonics, Billerica, MA, USA). Further analysis was performed using a MALDI-TOF spectrometer Ultraflex III (Bruker Daltonics, Billerica, MA, USA). The mass spectra of protein tryptic digests were recorded in a reflective positive ion mode in the 500–4200 m/z range. The mass spectra were processed and analyzed using the mMass v.5.5.0 software [22]. Database searches were performed against the NCBIprot database using the Mascot Peptide Mass Fingerprint service (http://www.matrixscience.com, accessed on 24 June 2021). Proteomic analysis for the PM16 phage has been described previously [18].

### 2.4. Animals

Three-month-old male CD-1 mice were obtained from the animal care facility in ICBFM, Novosibirsk. Animals were housed under a normal light-dark cycle; water and food were provided ad libitum. Outbred CD-1 mice were chosen to avoid the influence of the H2-haplotype on the results. Before experiments, feces from all involved mice were tested for the absence of phages specific to *P. mirabilis* CEMTC 73, *K. oxytoca* CEMTC 2927, *R. ornithinolytica* CEMTC 2544, *P. aeruginosa* CEMTC 1804, and *E. coli* TG1. A sample of feces from each mouse (~50 mg) was suspended in 2 mL of sterile PBS, pH 8.0 and clarified by centrifugation three times at 15,000× *g* for 10 min at 4 °C. After every centrifugation, the supernatants were transferred to new sterile tubes. Final supernatants were sterilized by filtration through a 0.22-μm filter (Millipore) and screened for phages specific to the above mentioned bacterial strains by spotting onto a fresh layer of each strain in the top agar. The plates were incubated overnight at 37 °C.

All animal procedures were carried out in accordance with the recommendations for the protection of animals used for scientific purposes (EU Directive 2010/63/EU). Immunized mice were euthanized via cervical dislocation. All experiments with animals were approved by the Inter-institutional Bioethics Committee of Institute of Cytology and Genetics Siberian Branch of Russian Academy of Sciences, Novosibirsk, Russia.

### 2.5. Endotoxin Quantification

Purified preparations of bacteriophages PM16, PM135, KP179, and PA136 were checked for endotoxin content using a Limulus amebocyte lysate (LAL) assay (Charles river laboratories Inc, Charleston, SC, USA) according to the manufacturer’s instructions.

### 2.6. Tissue Penetration Experiments

Five groups of mice (n = 12 in each group) were once administered an intraperitoneal injection of bacteriophages PM16, PM135, KP179, or PA136 at a target dose of 10^8^ PFU per mouse in 100 μL 0.9% NaCl. The sixth group of mice was administered the control phage M13 at the same dose. The control group of mice was injected with sterile 0.9% NaCl in a volume of 100 μL. Six hours after phage injection, blood samples were taken from six mice from each group; 24 h after injection, blood samples were taken from the remaining mice. A quantity of 100–150 μL of blood was collected into the chilled heparin-containing (100 IU) tubes by probing the retroorbital venous plexus with a glass capillary. From each blood sample, 15 μL was used to determine the titer of bacteriophages and the remainder of the blood sample was centrifuged at 4 °C and plasma was used to test the cytokine profiles. After euthanasia, brain, heart, lung, liver, kidney, and intestines were retrieved from sacrificed mice and 100 mg of each tissue was transferred to sterile tubes containing 100 μL 0.9% NaCl. Then, tissue samples were homogenized using a MagNA Lyser (Roche Diagnostics, Basel, Switzerland) immediately after harvesting, homogenates were centrifuged, and phage titers were determined in supernatants using a fresh layer of an appropriate bacterial host strain in the top agar. Phage titers in blood samples were determined simultaneously.

### 2.7. Determination of Cytokine Concentration in Plasma

The LEGENDplex Mouse Inflammation Panel (13-plex) (BioLegend, San Diego, CA, USA) was used to determine concentrations of cytokines in plasma; analysis was done according to the instructions of the manufacturer. Half of the animals in each group were withdrawn from the experiment at 6 h, and the remaining half at 24 h after phage injection. 

### 2.8. Phage-Specific Indirect ELISA of Sera of Immunized Mice

For immunization, four groups of mice (n = 30 in each group) were intraperitoneally administrated a selected phage (PM16, PM135, KP179, or PA136) at a target dose of 10^8^ PFU per mouse in 100 μL 0.9% NaCl. Each mouse was injected with a selected phage three times at one-week intervals with an equal amount of a phage without any adjuvants. Blood samples were collected before immunization and 1, 2, 4, 6, and 12 weeks after the first immunization, and serum samples were prepared. Individual serum samples from each mouse were used for ELISA. For further Western blotting and phage infectivity experiments, serum samples were pooled in an equal volume from each mouse according to anti-phage specificity and sampling time. 

To determine anti-phage IgG antibodies in mouse sera, the wells of 96-well plates (Medpolymer, Saint Petersburg, Russia) were coated with a selected phage (PM16, PM135, KP179, PA136, or M13K07) at the amount of 10^8^ PFU in the well in PBS, pH 8.0. After washing, each well was blocked with a 3% solution of skimmed milk in PBS, pH 8.0, with 0.1% Tween-20. Serial two-fold dilutions of appropriate anti-phage serum samples were added to the wells, starting from a 1:10 dilution, and incubated at 37 °C for 1 h. After washing, anti-mouse IgG peroxidase-conjugated rabbit polyclonal antibodies (Biosan, Novosibirsk, Russia) were added and incubated at 37 °C for 1 h followed by staining with 3,3’,5,5’-tetramethylbenzidine (Amresco, Solton, OH, USA). Antibody titers were calculated based on the crossing of dilution curves with the baseline [23]. Data were presented as relative titers, calculated as a ratio of different time point titers to the initial titer (0-week point) of the corresponding phage.

### 2.9. Western Blot Analysis

Purified phages were lysed with a buffer (50 mМ Tris-HCl, рН 6.8, 200 mМ dithiothreitol, 4% sodium dodecyl sulfate). Lysates were fractioned using 12% (*w*/*v*) SDS-PAGE and transferred onto a nitrocellulose membrane (Bio-Rad, Hercules, CA, USA). After blocking with 5% skim milk in PBS, pH 7.4 with 0.05% Tween, each membrane was cut into strips and each strip was incubated with the appropriate phage-specific serum diluted in PBS, pH 7.4 with 0.05% Tween, at 37 °C for 1 h. After washing, each strip was incubated with anti-mouse IgG or anti-mouse IgM peroxidase-conjugated rabbit polyclonal antibodies (Biosan) at 37 °C for 1 h followed by staining with 4-chloro-1-naphtol (Amresco).

### 2.10. Assessment of Phage Infectivity

Aliquots (20 μL in PBS) of serial dilutions of a selected phage were mixed with equal volumes of appropriate anti-phage pulled serum samples and incubated at room temperature for 1 h [24]. Then, each mixture was dropped on a fresh layer of the corresponding bacterial host strain in the top agar and plates were incubated at 37 °C overnight to reveal phage plaques. Phages mixed with non-immune mouse serum and phages mixed with PBS were used as controls. All experiments were done twice, each with two technical repeats.

### 2.11. Statistics

In tissue penetration experiments, the Kruskal–Wallis non-parametric test was used to compare the medians of the obtained PFU values. Differences between the groups were determined using the Dunn retrospective test. Statistical data were evaluated using Graph Pad Prism 7. The two-way multiple-measurement ANOVA test was used to compare ELISA data. The preliminary analysis of the distribution normality was performed using the D’Agostino and Pearson omnibus normality test.

## 3. Results

### 3.1. Mass-Spectrometry Analysis of Phage Structural Proteins

**Figure 1:**
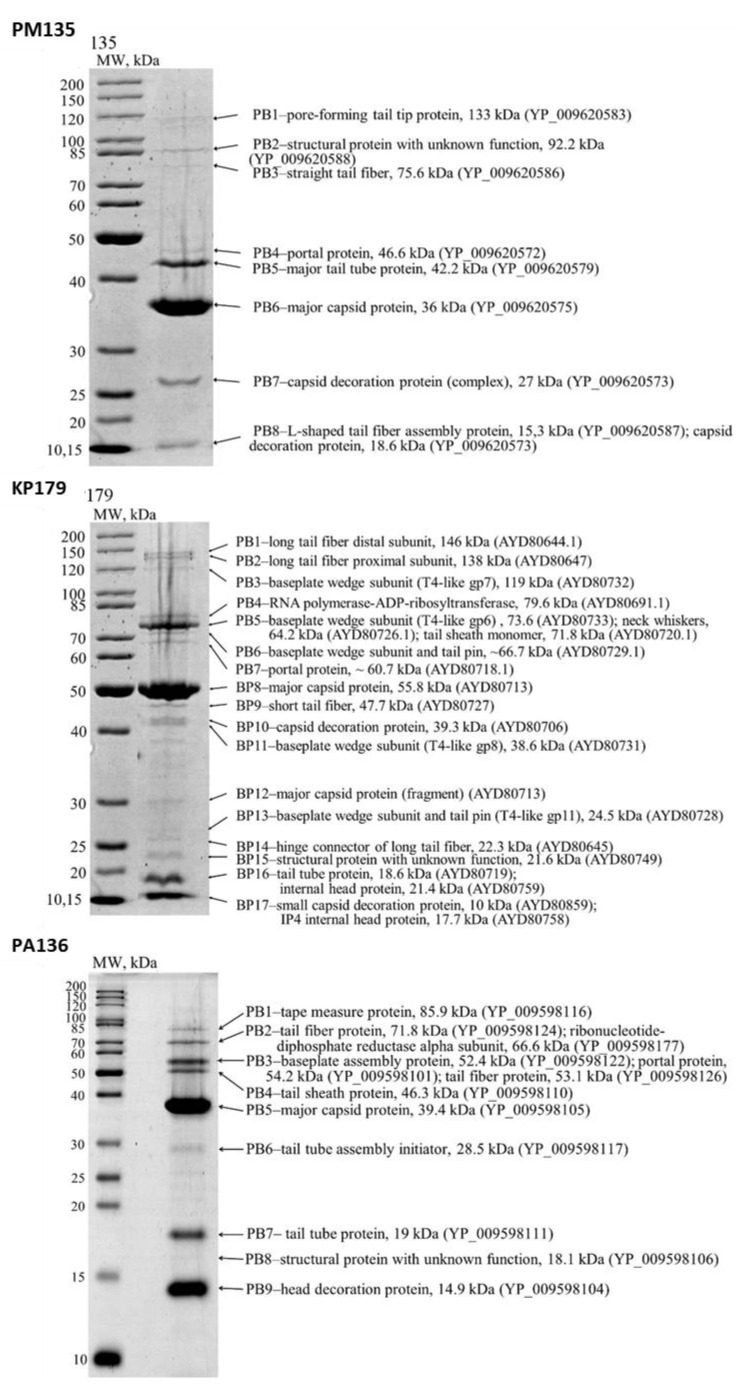
SDS-PAGEs of purified bacteriophage particles, followed by staining with Coomassie brilliant blue R250. The left lanes represent unstained protein standards (Thermo Fisher Scientific, Waltham, MA, USA). The names and molecular weights of the corresponding proteins are indicated on the right. GenBank identifiers for the sequences are shown in parentheses.

Nine, seventeen, and nine protein bands were revealed by SDS-PAGE for PM135, KP179, and PA136 phages, respectively (Figure 1). As a result of peptide mass finger-printing analysis [25], structural proteins from most bands with predicted functions were confirmed and open reading frames (ORF) encoding these proteins were determined (Figure 1). Ten, twenty-one, and eleven structural proteins of PM135, KP179, and PA136 phages, respectively, were identified. It should be noted that some bands corresponded to two or three phage proteins; however, peptide coverage was sufficient in all cases for reliable identification of all proteins with the predicted function. Mass-spectrometry analysis of phage PM16 structural proteins was performed previously [18] and twelve structural proteins with predicted functions were revealed, namely major capsid protein (37.5 kDa), two internal virion proteins (100.4 kDa and 22.7 kDa), internal core protein (140.1 kGa), head-tail connector protein (53.4 kDa), tail tubular protein A (20.7 kDa), tail tubular protein B (85.4 kDa), tail fiber protein (38.2 kDa), and four other virion proteins (~86 kDa, 77 kDa, 5.1 kDa, 9.8 kDa).

### 3.2. LAL Analysis of Phage Preparations

Serial dilutions of initial purified preparations of studied Caudovirales phages (10^11^–10^12^) in sterile 0.9% NaCl were checked for endotoxin content. Suspensions of phages PM16, PM135, KP179, PA136 at a concentration of 10^8^ PFU/mL showed substantial differences in endotoxin content: 0.5 EU/mL, 50 EU/mL, 0.5 EU/mL, and 5 EU/mL, respectively.

### 3.3. Bacteriophage Tissue Distribution

Purified preparations of phages PM16, PM135, KP179, PA136, and M13 were diluted in 0.9% NaCl to a concentration of 10^9^ PFU/mL and 100 μL of a phage suspension containing 10^8^ PFU of a distinct phage was used for administration to a mouse. 

Tissue distribution of phages PM16, PM135, KP179, PA136, and M13 was estimated based on their titers, which allowed detection of only viable phages. As equal amounts of the tissue samples were used, the number of estimated phages reflected their concentration in tissues. As 15 μL from each blood sample was used to determine the titer of bacteriophages, the obtained results were recalculated for 1 µL of blood. When phage tissue content is larger than that in blood samples, it may indicate phage accumulation in the tissue.

**Figure 2:**
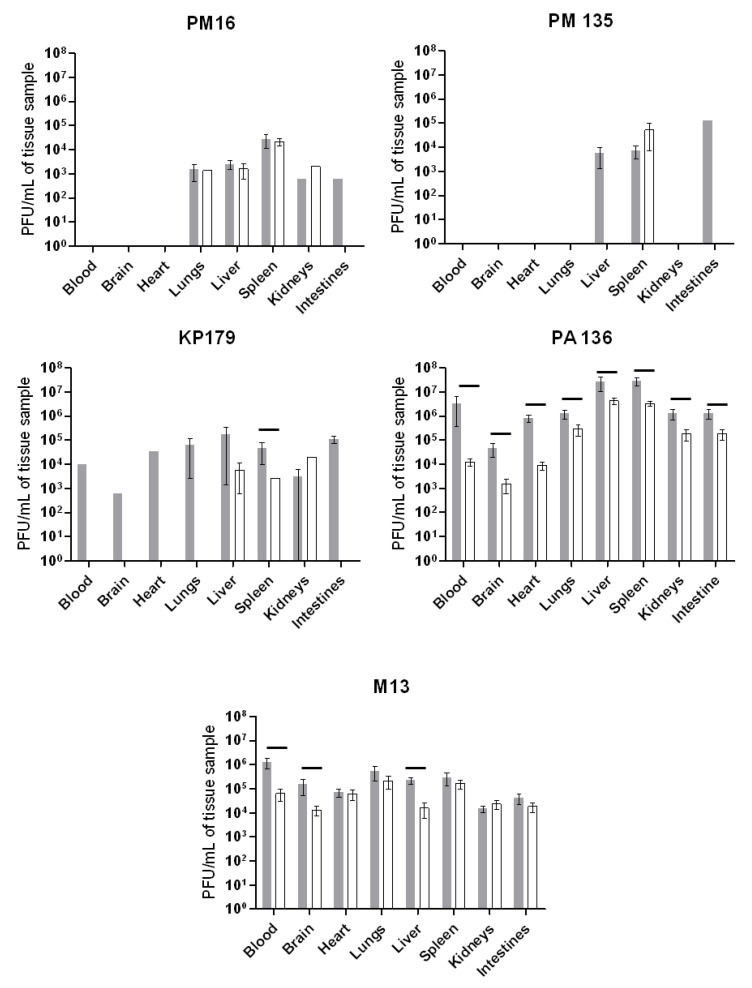
Tissue distribution of phages PM16, PM135, KP179, PA136, or M13 estimated based on their titers. Mice (n = 12 in each group) were once administered an intraperitoneal injection of a selected phage at a target dose of 10^8^ PFU per mouse in 100 μL 0.9% NaCl. Blood and tissue samples were taken from six mice from each group six hours after phage injection (grey columns) and 24 h after injection (white columns). Phage titers were determined using a fresh layer of an appropriate bacterial host strain in the top agar. Results are given in mean values ± standard deviation. Horizontal lines indicate statistical significance, *p* < 0.05.

The investigated bacteriophages revealed different patterns of tissue distribution (Figure 2). Both *P. mirabilis*-specific phages, PM16 and PM135, were undetectable in blood, brain, and heart at six and 24 h after their administration. The PM16 phage of the podovirus morphotype was found in the liver, lungs, kidneys, and intestine in similar titers, at 6 and 24 h, with the exception of the intestine, where it was not detected at 24 h. In the spleen, the titers of the phage PM16 exceeded the titers in the noted organs by at least ten times. The PM135 phage (siphovirus morphotype) was found in the spleen 6- and 24-h post-injection and in the liver, kidneys, and intestine of some mice at 6 h. Thus, both *P. mirabilis*-specific phages, PM16 and PM135, were able to accumulate in the spleen within 24 h of intraperitoneal injection in mice without any bacterial infection.

Tissue distribution of both phages of the *Myoviridae* family, KP179 and PA136, substantially differed. Viable KP179 particles were found in all screened tissues at 6 h and only in the liver, spleen, and kidneys with similar titers at 24 h after its administration. The PA136 phage was revealed in blood and all tested organs of mice, both at 6 and 24 h after injection, with the highest titers in the liver and spleen, and the lowest in the brain (Figure 2). In the liver and spleen, PA 136 titers were even higher than those for the M13 phage, whose good penetration into various organs had been shown previously [26,27].

Importantly, no viable phages specific to the bacterial strains *P. mirabilis* CEMTC 73, *K. oxytoca* CEMTC 2927, *R. ornithinolytica* CEMTC 2544, *P. aeruginosa* CEMTC 1804, and *E. coli* TG1 that are hosts for phages PM16, PM135, KP179, PA136, and M13 were found in blood and all tested organs of mice from a control group. These results confirmed that both the examined phages and other phages specific to these bacterial strains were absent in mice. 

### 3.4. Serum Cytokine Profiles at 6 and 24 h after Phage Introduction

**Figure 3:**
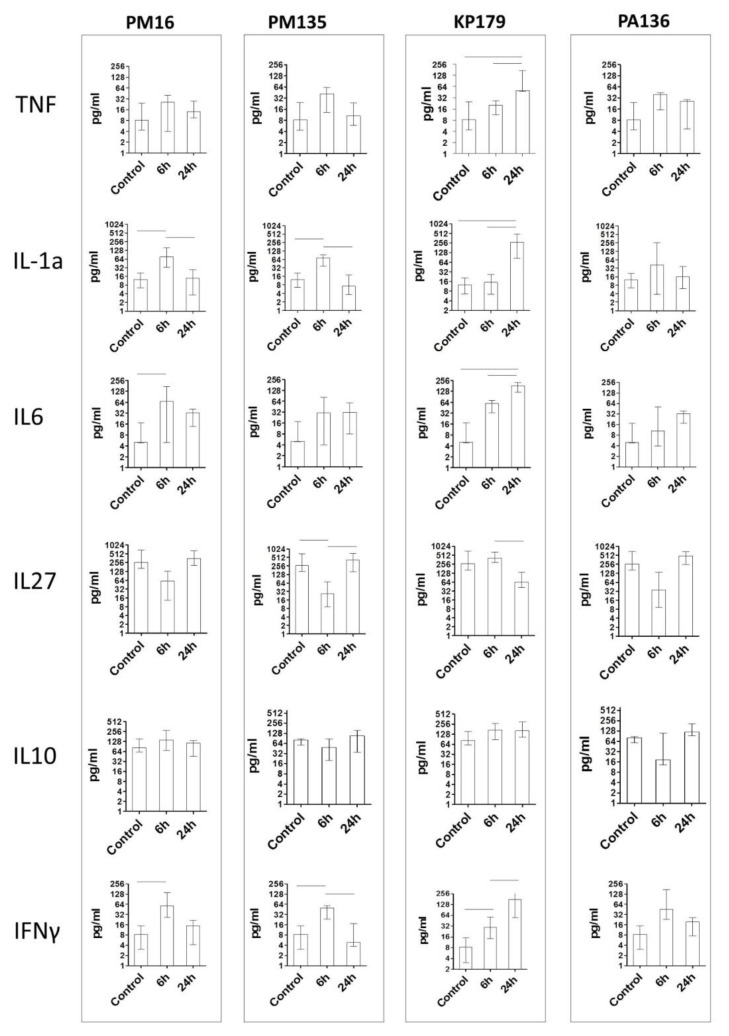
Cytokine concentration in plasma of mice once administered an intraperitoneal injection of the phages at a target dose of 10^8^ PFU per mouse in 100 μL 0.9% NaCl. Control indicates injection of 100 μL 0.9% NaCl. Cytokine concentrations were evaluated six and 24 h after phage injection. Results are given in medians with interquartile range. Horizontal lines indicate statistical significance, *p* < 0.05.

The PM16 phage induced a remarkable increase in IL-1, IFNg, and IL-6 levels at six hours after phage administration, followed by their decrease at 24 h (Figure 3), whereas IL-27, in contrast, showed a more than two-fold decrease in its median level at six hours and increased its initial level at 24 h after phage introduction. IL-10 showed a less prominent elevation. The PM135 phage demonstrated a similar pattern for the same set of cytokines, showing only minor differences in the cytokine levels. PA136 injection was accompanied by similar patterns for IL-1, IFNg, IL-6, and IL-27, but not for IL-10, which was downregulated at 6 h after phage administration with subsequent normalization at 24 h (Figure 3).

Changes in cytokine levels after administration of the KP179 phage differed from those of the other tested phages. Thus, IL-1 demonstrated retarded elevation, with its level being more than twice that of other phages at 24 h. IFNg and IL-6 showed gradual growth and increased their levels more than twice compared to those of other phages. The IL-27 level demonstrated a retarded decrease at six hours, and its level was only reduced at 24 h; IL-10 showed no striking difference in its time pattern compared to the other phages. In all immunized groups, IL-1, IFNg, and IL-6 showed hump-like behavior, reflecting transient elevation at 6 h; however, KP179 induced elevation of these cytokines for a longer period. IL-27 also differed substantially from KP179 and the other phages (Figure 3).

It should be noted, that despite the marked differences in endotoxin content in phage preparations, no association was found between endotoxin levels and serum cytokine profiles.

### 3.5. Analysis of Anti-Phage Serum Immunoglobulins

**Figure 4:**
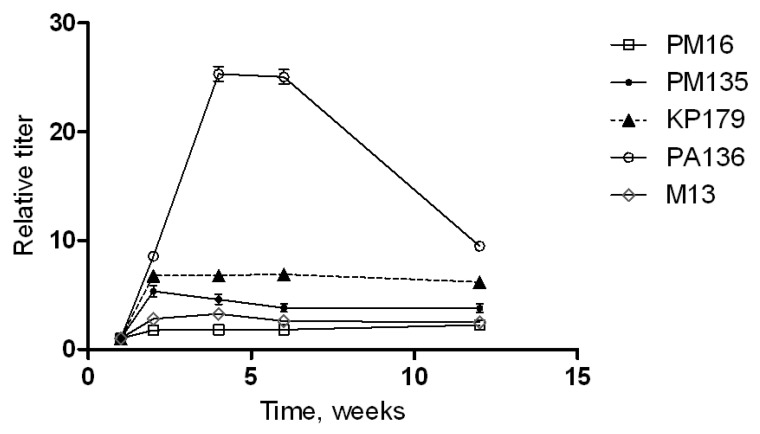
Phage-specific indirect ELISA of sera of immunized mice. Serum samples were obtained before immunization and 1, 2, 4, 6, and 12 weeks after the last immunization of mice injected three times with a selected phage (PM16, PM135, KP179, PA136, or M13) without any adjuvants. Antibody titers were calculated based on the crossing of dilution curves with the baseline. Results are given as relative antibody titers, calculated as a ratio of different time point titers to the initial titer (before immunization) of antibodies against the corresponding phage.

Changes in anti-phage serum polyclonal IgG antibodies appearing after immunization of CD1-mice with phages without any adjuvant were evaluated by ELISA (Figure 4). Different patterns were detected for the investigated phages. No significant increase in anti-PM16 IgG level was revealed. Another *P. mirabilis*-specific phage, PM135, showed a five-fold increase in IgG levels two weeks after the first immunization, followed by a gradual decrease in anti-PM135 IgG levels in mouse serum. Similar growth was recorded for anti-KP179 IgG at two weeks after the first immunization; however, elevated levels of these antibodies remained for at least 12 weeks (Figure 4).

The most significant growth of relative titers was observed for PA136-specific IgG. A 25-fold increase in these antibodies was found at 4–6 weeks after the first immunization and a rapid decrease in the anti-PA136 IgG level over the following six weeks (Figure 4). These results indicate the high immunogenicity of the PA136 phage for the mice immune system.

### 3.6. Western Blot Analysis

Western blot analysis was performed to identify immunogenic phage proteins recognized by IgM and IgG antibodies from mouse sera collected before immunization and 1, 2, 4, 6, and 12 weeks after immunization of CD1-mice with a corresponding phage without adjuvant. Bands for PM135, KP179, and PA136, but not for PM16, were detectable. The absence of protein bands of PM16 phage coincided with the results of ELISA and confirmed low immunogenicity of this phage administrated to CD1 mice without any adjuvant.

**Figure 5:**
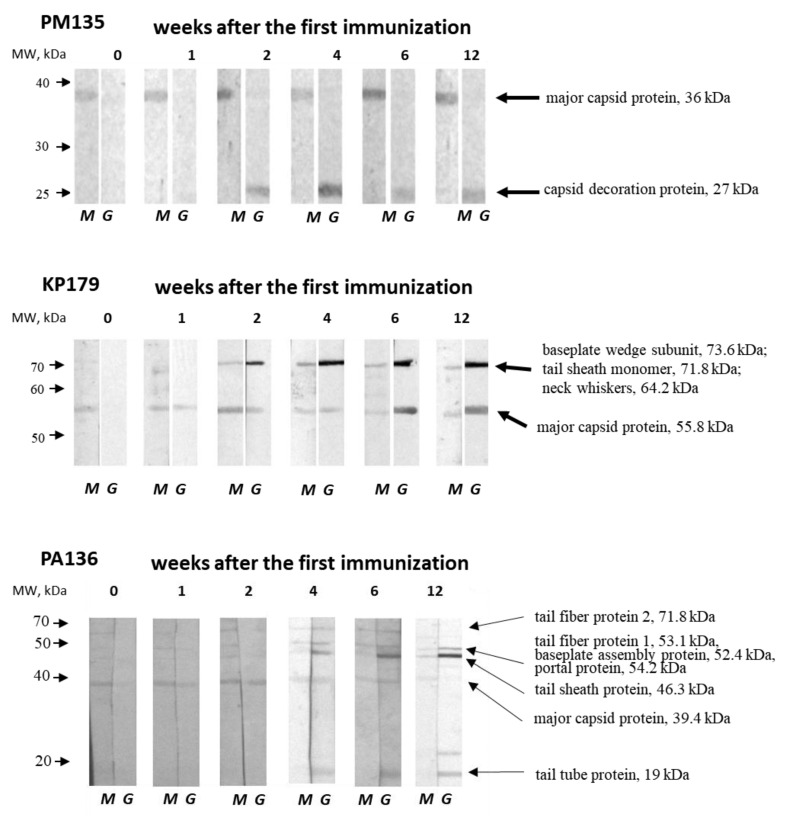
Western blot analysis of phage lysates fractioned using 12% SDS-PAGE and transferred onto a nitrocellulose membrane (Bio-Rad, USA). Phage proteins were revealed by anti-phage IgM (M) and IgG (G) antibodies from pooled serum samples obtained before immunization and 1, 2, 4, 6, and 12 weeks after the first immunization of mice injected three times with a selected phage (PM135, KP179, or PA136) without any adjuvants. The left arrows correspond to molecular weights of protein standards (Thermo Fisher Scientific, USA). The names and molecular weights of the identified proteins are indicated on the right.

One PM135 protein of approximately 35 kDa was revealed by IgM from sera obtained both before and after immunization (Figure 5). Mass spectrometry showed that the protein corresponds to the mature major capsid protein (36 kDa). IgG antibodies against another protein of over 25 kDa appeared two weeks after the first immunization with PM135 (Figure 5). Mass spectrometry showed that this protein corresponds to the capsid decoration protein (~27 kDa) of PM135.

Two bands corresponding to immunogenic KP179 proteins were identified by both IgM and IgG from KP179-specific serum samples (Figure 5). The first protein band of slightly more than 50 kDa was recognized by IgM from intact and immunized mice, and was detectable by IgG one week after the first immunization. The second protein band, with a molecular weight between 70 and 80 kDa, was visible at two weeks after immunization using both IgM and IgG. The intensity of both bands revealed by IgG steadily increased, reaching a maximum at four–six weeks, which coincides with the results of ELISA. Mass spectrometry indicated that the first protein band matches well with the KP179 major capsid protein (55.8 kDa); however, several KP179 proteins were identified in the second protein band —tail-sheath monomer (71.8 kDa), baseplate wedge subunit (73.6 kDa), and neck whiskers protein (fibritin) (64.2 kDa). The tail-sheath protein is the most probable immunogen due to its copy number. 

IgM antibodies from intact mice were able to bind to a PA136 protein of ~40 kDa, which is likely to be a PA136 capsid protein (39.4 kDa) according to mass spectrometry (Figure 1 and Figure 5). This protein was recognized by IgM and IgG from PA136-specific serum samples, with the exception of IgG from sera collected 12 weeks after the first immunization. A diversity of protein bands were revealed by IgM and IgG from specific serum samples starting from the second and fourth weeks after immunization, respectively (Figure 5). PA136 proteins detected by IgM may correspond to tail fiber protein 1 (53 kDa) and tail fiber protein 2 (71.8 kDa). IgG probably revealed PA136 tail fiber protein 1 (53 kDa), putative baseplate assembly protein and/or portal protein (52.4 and/or 54.2 kDa), tail sheath protein (46.3 kDa), and tail tube protein (19 kDa). The changes in the intensity of individual bands coincide with the results of ELISA with PA136-specific sera.

Notably, IgM-class antibodies against major capsid proteins of PM135, KP179, and PA136 phages, but not PM16, were found in sera of intact mice. The intensity of the corresponding protein bands did not increase after immunization. These pre-existing IgM antibodies might indicate previous contact of mice with these phages. However, the absence of phages specific to the host bacterial strains (*P. mirabilis* CEMTC 73, *K. oxytoca* CEMTC 2927, *R. ornithinolytica* CEMTC 2544, and *P. aeruginosa* CEMTC 1804) and phages in fecal samples of the mice was tested prior to the experiments. 

### 3.7. Influence of Immune Antisera on Phage Infectivity

**Figure 6:**
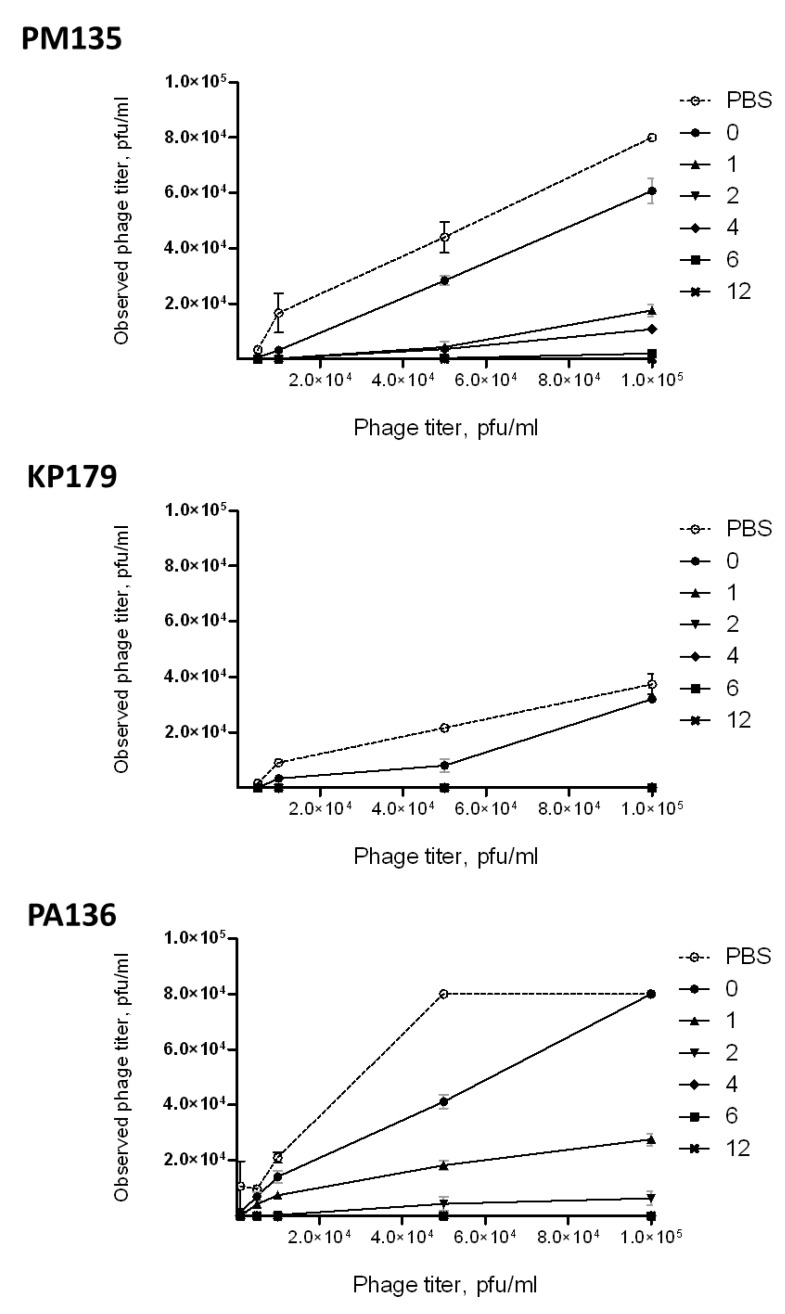
Neutralizing activities of pooled serum samples obtained before immunization and 1, 2, 4, 6, and 12 weeks after the last immunization of mice intraperitoneally injected three times with a selected phage (PM135, KP179, or PA136) without any adjuvants.

No changes in phage infectivity were detected for anti-PM16 mouse serum samples. However, when lytic activities of phages PM135, KP179, or PA136 treated with serum collected after immunization were compared with those for the phages mixed with sera obtained before immunization, a substantial decrease in phage lytic activities was found (Figure 6). For PM135 and PA136, immune sera collected 4, 6, and 12 weeks after the first immunization eliminated lytic activities of the corresponding phages. Moreover, phage infectivity of KP179 was fully inhibited by sera derived starting from the first week after immunization. The obtained results indicated that antibodies elicited by PM135, KP179, or PA136 phages can neutralize infectivity of these phages. Notably, serum samples collected before administration of PM135 or KP179 exhibited the ability to reduce phage infectivity compared to PBS (Figure 6). This result corresponds well with the identification of pre-existing IgM in serum samples from mice before immunization.

## 4. Discussion

Despite the relative simplicity of the idea of phage therapy, a large number of questions remain to be answered. In this study, we compared phages of different sizes and morphotypes in terms of their tissue distribution and ability to influence the humoral immunity. Four distinct Caudovirales bacteriophages were involved: two *P. mirabilis* phages PM16 and PM135 (the same host strain, podovirus and siphovirus morphotypes, respectively), and two phages from the *Myoviridae* family specific to different hosts—*Klebsiella* spp. phage KP179, and *P. aeruginosa* phage PA136. Our previous studies have shown that PM16 and PM135 have a narrow host range; KP179 and PA136 can infect a lot of bacterial strains [18,19]. PM16, PM135, KP179, and PA136 phages demonstrated high lytic activities; complete genome sequencing confirmed the absence of the genes encoding toxins or associated with lysogeny. Thus, these phages might be potentially used in phage therapy. 

When both the ability of phages to penetrate tissues [27] and phage immunogenicity [24,26] had been studied, no clear association between two phenomena had been discussed. In this study, we demonstrated that the higher presence of bacteriophages in tissues, particularly in spleen and liver, is associated with increased phage immunogenicity provided by phage-specific antibodies with neutralizing properties. We elucidated phage distribution throughout murine tissues after intraperitoneal injection. There was no observable universal pattern of bacteriophages’ tissue distribution and retention. Thus, while the PA136 and M13 phages were found in all examined mouse tissues, both at 6 and 24 h after administration, the others (PM16, PM135, and KP179) were undetectable in blood and some organs at 24 and sometimes 6 h. It was found that phages were most present in the spleen, liver, and lungs with their titers in these organs were up to ten-fold higher than in other organs and blood. This observation is in a good agreement with the results of the previous study [10].

Bacteriophage presence in tissues is defined by the difference between phage accumulation and clearance. The accumulation depends on bacteriophage capacity to be captured by mammalian cells, the feature that is mainly referred to cellular uptake via micropinocytosis and transcytosis [28]. The size of bacteriophage particles as it has been shown in in vitro experiments is one of the important factors with larger particles to be less permeable [28]. The clearance is believed to be exerted by immune cells, such as macrophages and B-cells [29,30] with no specific ligand-receptor interactions being found yet. However, we found no evidence providing phages to be presented in tissues in connection with their sizes that coincides with the results of previous in vivo studies [26,31]. Nevertheless, some taxonomy dependence was evaluated as *Myoviridae* phages (KP179 and PA136) were presented in tissues in higher titers than other tailed phages (PM16 and PM135, both having the same *P. mirabilis* host). However, this observation requires strong confirmation with a number of phages of different morphology. 

The obtained data on serum cytokine profiles after phage introduction confirmed the ability of phages to induce cytokine production. First, it stimulates non-adaptive cytokines such as TNF and IL1a, indicating macrophage involvement. Second, the early increase in IFNg and IL27 levels, which are the hallmark of adaptive immune response, suggests the involvement of already present phage-specific T or B cells. The elevation of pro-inflammatory cytokine levels for most of the examined phages was transient, lasting approximately 24 h, with only KP179 showing a delayed and more prominent increase in levels of blood cytokines.

As endotoxins are common in Gram-negative phage preparations, it is possible that cytokine response just reflects the quality of the purification step [32,33]. However, the level of endotoxin detected in current study did not correlate with phage immunogenicity, retention, and cytokine elevation. Endotoxin presence may only explain TNF and IL1a elevation, while interferon response seems to be the reaction on the bacteriophage DNA as it has been previously shown [34]. 

What is more intriguing is that the comparison of phage tissue accumulation and features of humoral immune response demonstrates clear association. Thus, phage presence in spleen and liver, as well as ELISA IgG titers after immunization increased in the order PM16 < PM135 < KP179 < PA136.

An important finding in our study was the identification of pre-existing IgM-class antibodies directed against capsid proteins of phages of myovirus and siphovirus morphotypes. Although it is uncommon to detect IgM during the secondary immune response, a rapid increase in IgG levels after initial immunization may indicate previous contact of the experimental mice with the phages. However, taking into consideration the absence of phages specific to host bacterial strains (*P. mirabilis* CEMTC 73, *K. oxytoca* CEMTC 2927, *R. ornithinolytica* CEMTC 2544, and *P. aeruginosa* CEMTC 1804) in fecal samples from mice before the experiments, we can hypothesize that pre-existing IgM antibodies belong to the primary (naive) repertoire that provides initial recognition of phages. Another hypothesis is that these IgM antibodies are cross-reactive and directed against similar epitopes exposed on the surface of related phages from normal mouse microbiota. This hypothesis is supported by data of multiple alignment analysis, which has indicated homology between similar proteins of different phages [35]. Phages of the normal mice microbiome may yield proteins similar to proteins of other (external) phages. These proteins obviously have no capacity to stimulate IgG class switching but serve as a source for T-cell-independent IgM diversity. 

IgM-class antibodies could partially explain both tissue distribution patterns and immunogenicity, demonstrated by the rapid increase in IgG levels after immunization. The retention might be associated with IgM-dependent immune complex formation that tends to accumulate in such compartment as pleural cavity, kidneys, and pericardium. On the other hand, some phage properties increasing their retention might influence immunogenicity drastically. Thus, retention of protein or viral particles by follicular dendritic cells (FDC) increases the efficiency of antibody class switching [36]. Indeed, PA136 being the most retained phage, demonstrated the most prominent capacity of IgM to IgG-class switching in our study. Both mechanisms can be tightly related because FDCs require IgM-dependent retention of proteins to present them to B-cells. However, as phages of Caudovirales order are known to bear immunoglobulin-like domains [37], the direct ligand-receptor interactions between immune cells and phages are also possible, though have not been shown yet. 

Several conclusions can be made based on the obtained results. First, tissue distribution of bacteriophages appears to be dependent on specific proteins rather than the capsid morphotype. Second, regardless of the taxonomy, phages are unable to stimulate a substantial and long-lasting increase in pro-inflammatory cytokines, due to the initial interaction with microorganism tissues. Third, bacteriophages can stimulate adaptive humoral immune response in accordance with their spleen- and liver-retention capacity. It seems that the higher retention may provide the higher antibody titers. This should be considered during development of personalized therapeutic strategies because pre-existing phage-neutralizing activity may develop early and significantly worsen the outcome [12]. Thus, although phage therapy appears to be a powerful means of fighting infectious diseases, further understanding is necessary before phage therapy can be considered a viable approach.

## Figures and Tables

**Table 1:** Characteristics of the investigated phages.

Phage	Family/ Genus	Morphotype	Capsid Diameter, nm	Tube Length, nm	Genome Size
PM16	*Autographiviridae*/*Novosibovirus*	podovirus	50	15	41,268 bp
PM135	*Demerecviridae*/*Novosibvirus*	siphovirus	75	162	104,329 bp
KP179	*Myoviridae*/*Jiaodavirus*	myovirus	92	87	162,630 bp
PA136	*Myoviridae*/*Pakpunavirus*	myovirus	65	91	92,037 bp
M13K07	*Inoviridae*/ *Inovirus*	filamentous	No capsid	800 (length of filament)	8668 bp
